# Effects of Spiciness Intensity on Emotional Responses to Xianglajiang: A Comparison Between Korean and Chinese Consumers

**DOI:** 10.3390/foods15193401

**Published:** 2026-09-24

**Authors:** Insun Kang, Binglin Liu, Min A Lee

**Affiliations:** 1 Department of Foods and Nutrition, Kookmin University, Seoul 02707, Republic of Korea; inseon2450@kookmin.ac.kr; 2 College of Cooking Science and Technology, Jiangsu College of Tourism, Yangzhou 225000, China

**Keywords:** spiciness, emotional response, Xianglajiang, sensory evaluation, consumer acceptance

## Abstract

This study explored emotional responses to three levels of spiciness in Xianglajiang, a traditional Chinese spicy sauce, while examining the effects of nationality and sensation-seeking tendencies. A total of 111 participants (61 Koreans and 50 Chinese) evaluated Xianglajiang samples with mild, medium, and high spiciness levels. Emotional responses were assessed using a Rate-All-That-Apply questionnaire, alongside measures of sensation-seeking tendencies, preferences, attitudes, and purchase intentions. Data were analyzed using two-way mixed ANOVA, chi-square analyses, PCA and correlation analysis. The results indicated that negative emotions significantly increased with spiciness intensity, while positive emotions remained relatively stable. Korean consumers reported higher levels of negative emotions across all spiciness conditions than Chinese consumers, despite both groups exhibiting a notable rise in negative affect as spiciness intensified. Significant cross-national differences emerged in several emotional terms, including curious, overjoyed, surprised, fearful, nervous, pained, and uncomfortable. Principal component analysis revealed distinct emotional response patterns based on nationality and spiciness level. Additionally, Chinese participants displayed significantly higher sensation-seeking tendencies than their Korean counterparts. Positive emotions positively correlated with preference, attitude, and purchase intention, while negative emotions exhibited negative associations. These findings indicate that emotional responses to spicy foods are influenced by spiciness intensity, cultural familiarity, and sensation-seeking tendencies, offering insights for the development and localization of spicy food products.

## 1. Introduction

Taste perception is essential in shaping consumers’ food choices and acceptance [1]. It is typically classified into five basic categories: sour, sweet, salty, bitter, and umami [2]. While spiciness is not considered a basic taste but rather a chemesthetic sensation arising from trigeminal nerve stimulation [3,4], it is recognized as a vital aspect of the overall sensory food experience [5].

In our globalized world, traditional foods are increasingly accepted across different cultures [6]. Media, travel, and migration have facilitated consumers’ exposure to diverse cuisines, enhancing interest in new flavors and extending traditional foods beyond their regions of origin [7]. These foods also serve as important markers of national identity and cultural value [8]. Consequently, several governments actively promote the globalization of their traditional cuisines, emphasizing the need to understand foreign consumers’ acceptance of these foods and taste preferences [9].

Consumers play a crucial role in sensory and consumer science as both recipients and evaluators of food [10]. Individual characteristics, along with repeated exposure, significantly shape food familiarity and preferences [11,12]. Prior studies have explored the relationship between familiarity and preference for Korean traditional foods among various national groups. For instance, a study [13] found that Korean consumers perceived Yakgwa as both the most familiar and preferred product, followed by Japanese and French consumers. These findings suggest that national familiarity significantly influences consumer acceptance of traditional foods across different cultures. Another study [14] revealed that emotional responses and preferences for salad dressings and dipping sauces based on traditional Korean sauces varied considerably among consumers from Korea, the United States, and the United Arab Emirates. These insights indicate that assessing food-evoked emotions can enhance our understanding of food consumption and consumer behaviors in a multinational context. Previous studies have demonstrated that food-evoked emotions are important predictors of consumer behavior, including food liking, purchase intention, and product acceptance. Therefore, evaluating food-evoked emotions provides valuable information beyond traditional hedonic measures [15].

As Asia’s economy has grown, its culture, particularly food culture, has gained global prominence [16]. However, cross-national research on traditional foods remains limited. Sauces enhance flavor, texture, and viscosity while reflecting national identity [17,18,19]. The Chinese sauce Xianglajiang, recognized for its deep aroma and spiciness, is a staple condiment from the Sichuan region. It is typically prepared with chili peppers and spices such as huajiao (Sichuan pepper) and is utilized in various forms, including sauces and seasonings [20]. In addition to these ingredients, Xianglajiang is commonly prepared with black pepper, sesame, vegetable oil, and natural spices. It is characterized by its aromatic, numbing, spicy, and savory taste with a lingering aftertaste. The production process combines the traditional preparation method of douhua dipping sauce with modern preservation technologies, resulting in a variety of products, including stir-fried minced meat sauce, nut-enriched sauce, and beef chili sauce. Typical ingredients include fermented black soybeans (douchi), tomato paste, and lean minced meat, while high-temperature stir-frying imparts the sauce with its characteristic rich aroma, bright red appearance, and intense flavor [21]. Familiar to Chinese consumers, Xianglajiang serves as an ideal stimulus for investigating how national familiarity influences emotional responses and preference formation. In addition, its unique flavor profile may lead to variations in perceived pungency and overall sensory experience, even at comparable spiciness levels. Previous studies have shown that increasing spiciness intensity influences consumer acceptance and emotional responses. Moderate spiciness may enhance food enjoyment, whereas excessive spiciness can induce discomfort and negative emotions. However, these findings have mainly been reported for specific spicy foods, and evidence regarding traditional Chinese spicy sauces remains limited [22].

Spiciness intensity extends beyond a mere sensory attribute; it can significantly influence consumers’ emotional responses, eliciting both positive and negative feelings. The nature and intensity of these emotional reactions may vary based on the level of spiciness and the consumers’ national backgrounds. In particular, consumers may respond more positively to sauces that are culturally familiar. Nevertheless, previous studies have primarily focused on specific spicy foods or isolated national contexts, resulting in a limited understanding of how consumers from diverse backgrounds emotionally react to spicy sauces with varying levels of spiciness. Despite the rising global appeal of Chinese cuisine, research comparing emotional responses to spicy sauces across different national backgrounds remains scarce. Since emotions can influence various consumer behaviors, including purchase intentions, exploring these emotional differences by national background may provide valuable insights for global food product development and localization strategies. Furthermore, even at similar spiciness levels, emotional responses may differ based on flavor characteristics and cultural familiarity. Therefore, further research is warranted to examine emotional responses to traditional spicy sauces across varying spiciness intensities while considering cultural familiarity.

Beyond national familiarity, individual personality traits may also shape consumers’ reactions to spicy foods. The sensation-seeking tendency, a psychological trait characterized by a preference for novel, complex, and intense stimuli [23,24], has been linked to food choices and preferences for spicy foods. Individuals with high sensation-seeking tendencies typically display a greater preference for and consumption of chili peppers, suggesting that they derive pleasure from spicy stimulation [11]. Consequently, emotional responses to the same spicy stimulus may vary according to individual personality traits, indicating that sensation-seeking tendency should be considered alongside cultural background as a key factor influencing the acceptance of spicy foods.

Collectively, previous studies have demonstrated that spiciness intensity, national background, and sensation-seeking tendencies each influence consumer responses to spicy foods. However, these factors have rarely been investigated simultaneously in the context of traditional Chinese spicy sauces. Furthermore, little is known about how emotional responses to different levels of spiciness vary across consumers from different national backgrounds. Therefore, the present study was designed to address these gaps by examining emotional responses to Xianglajiang with varying spiciness levels among Korean and Chinese consumers.

This study aimed to investigate emotional responses to varying levels of spiciness in Xianglajiang and to compare these responses between Korean and Chinese consumers. Based on previous research on food-evoked emotions, cultural background, and sensation-seeking tendencies, we hypothesized that (H1) increasing spiciness intensity would elicit distinct emotional responses, particularly stronger negative emotions; (H2) Korean and Chinese consumers would exhibit different emotional response patterns toward Xianglajiang; (H3) positive emotions would be positively associated with consumer acceptance (preference, attitude, and purchase intention), whereas negative emotions would be negatively associated with these consumer acceptance measures; and (H4) Korean and Chinese consumers would differ in sensation-seeking tendencies, providing additional insight into cross-national differences in consumer responses to spicy foods. Ultimately, this study sought to enhance the understanding of cross-national emotional responses and consumer acceptance of a traditional Chinese spicy sauce across varying spiciness levels. The findings are expected to provide practical implications for the development and localization of spicy food products in global markets.

## 2. Materials and Methods

### 2.1. Participants

Participants aged 19–59 years were recruited through online and offline methods. Online recruitment utilized internet communities and social media platforms, while offline efforts involved posting advertisements on campus bulletin boards. Participants were selected based on predefined inclusion and exclusion criteria. The exclusion criteria included smokers, individuals who were pregnant or potentially pregnant, and those taking medications affecting taste perception. Sensation-seeking tendencies were assessed using a modified version of the Sensation Seeking Scale [25], tailored to reflect dietary behaviors and preferences for spicy foods. The scale comprised four subdimensions: Thrill and Adventure Seeking (TAS), Experience Seeking (ES), Disinhibition (DIS), and Boredom Susceptibility (BS), each containing three items, resulting in a total of 12 items rated on a 5-point Likert scale. The modified scale was designed to assess sensation-seeking tendencies in the context of food-related behaviors rather than general sensation-seeking. The modified scale demonstrated acceptable internal consistency (Cronbach’s α = 0.877).

Sensory evaluations were conducted separately in South Korea and China. The same experimental protocol, including sample preparation, serving conditions, evaluation procedures, and questionnaire administration, was applied at both locations to ensure consistency across study sites.

In total, 113 responses were collected, with 111 included in the final analysis after excluding two incomplete or insincere responses. The final sample comprised 61 Korean Participants and 50 Chinese participants, with sex distribution controlled for comparability. This study received approval from the Institutional Review Board of Kookmin University (Approval No.: KMU-202601-HR-545). 

### 2.2. Spicy Sauce Samples

Potatoes were selected as the medium for the experimental samples owing to their common consumption in both Korea and China and their uniform physical properties, which allowed for controlled manipulation of spiciness intensity and consistent comparison across stimuli. The potatoes were cut into 1 cm^3^ cubes, boiled in 500 mL of distilled water, and portioned into 70 mL sample cups at approximately 20 g per serving. To standardize spiciness intensity, commercially available Xianglajiang products with defined pungency levels were utilized. Xianglajiang from the Dezhuang Group (Li Spicy Unit levels: 45, 52, and 65; corresponding to contents of main capsaicinoid (CMC) ranges of 94.0–189, 201–789 and 596–1351 mg/kg, respectively) served as the experimental stimulus, establishing three spiciness conditions (mild, medium, and high) based on manufacturer-provided pungency information. Each sauce sample was portioned at 7 g into separate 70 mL cups. All ingredients were purchased online. To ensure consistency, preparation and portioning occurred on the day of the experiment, with all samples stored at room temperature prior to evaluation.

### 2.3. Measurements

Food-evoked emotions in response to spiciness were assessed using the lexicon developed by Ren et al. [26], which comprises 33 emotion descriptors—21 positive and 12 negative—specifically designed for spicy foods. Sample preferences were evaluated across five attributes: appearance, aroma, taste, texture, and overall preference, employing a 5-point scale (1 = “extremely dislike” and 5 = “extremely like”). Attitude and purchase intention were each measured using four items on a 5-point Likert scale (1 = “strongly disagree” and 5 = “strongly agree”). The internal consistency values for both measures were acceptable (Cronbach’s α = 0.883–0.935 for attitude and 0.882–0.945 for purchase intention). The questionnaire was developed in both Korean and Chinese, with a bilingual co-researcher ensuring linguistic equivalence through translation and back-translation procedures.

### 2.4. Procedure

This study was conducted in two sessions at 10:00 a.m. and 2:00 p.m., each lasting approximately 60 min. Upon arrival, participants received a 5-min briefing on the study procedures before commencing the tasting according to the provided instructions. Food-evoked emotions were assessed using the Rate-All-That-Apply (RATA) method, with the intensity of each selected emotion rated on a 5-point scale (1 = “very weak” and 5 = “very strong”). Samples were presented in containers labeled with random three-digit codes and served in a randomized order under blind testing conditions. After tasting each sample, participants sequentially evaluated their emotional responses, preferences, attitudes, and purchase intentions. To minimize carryover effects, participants rinsed their mouths with room-temperature water and unsalted crackers between samples, with a 5-min interval between sample presentations to prevent sensory fatigue.

### 2.5. Data Analysis

Chi-square (χ^2^) tests and independent samples *t*-tests were conducted to examine differences in demographic characteristics and spicy food consumption patterns between Korean and Chinese participants. For each participant, the aggregate positive and negative emotion scores were calculated as the mean ratings of the 21 positive and 12 negative emotion descriptors, respectively. This approach was used to represent the overall level of positive and negative emotional responses while reducing the complexity of analyzing individual emotion descriptors separately. Aggregate positive and negative emotion scores were subjected to analysis of variance (ANOVA), while individual emotion descriptor frequencies were used for chi-square analyses and principal component analysis (PCA) visualization. A two-way mixed ANOVA was employed to determine whether emotional responses varied by spiciness intensity across nationalities, with spiciness level as the within-subjects factor and nationality as the between-subjects factor. The assumption of sphericity was tested, and Greenhouse–Geisser corrections were applied as needed. Partial eta squared (ηp^2^) was reported as the measure of effect size for the mixed ANOVA results. In addition, chi-square analyses were performed to examine differences in the selection frequency of individual emotion descriptors by nationality across spiciness levels. To mitigate Type I error from multiple comparisons, the Bonferroni correction adjusted the significance level from 0.05 to *p* < 0.0015 for the 33 repeated comparisons.

Preference, attitude, and purchase intention scores were analyzed to identify differences based on spiciness intensity and nationality. PCA and biplot visualization of emotional response patterns were performed using R Studio 4.6.0. PCA was conducted separately for Korean and Chinese participants using the original frequencies of the selected emotion terms without additional preprocessing or scaling. Biplots were generated to visualize the relationships between the three spiciness levels and individual emotion descriptors within each nationality. All other analyses were conducted using IBM SPSS Statistics 26.0.

## 3. Results

### 3.1. Demographics of Study Participants

Table 1 presents the general characteristics of the participants. In total, 111 participants (61 Koreans and 50 Chinese) completed the study. No significant differences were found between the Korean and Chinese groups regarding age, occupation, or frequency of spicy sauce consumption. However, significant differences emerged in education, preference for spicy sauces, and sensation-seeking.

### 3.2. Emotional Response Frequencies and PCA Biplot for Xianglajiang

Chi-square analyses were conducted to ascertain whether the selection of specific emotion descriptors varied significantly between Korean and Chinese participants across different spiciness levels (mild, medium, and high). To account for multiple comparisons, Bonferroni correction was applied, setting the adjusted significance level at *p* < 0.0015.

In addition, PCA was performed to visually explore the relationships between emotion descriptors and spiciness intensity levels, with results presented as biplots.

For Xianglajiang, notable differences in emotional responses were observed between Korean and Chinese participants across varying spiciness levels (Table 2). In the mild condition, Korean participants reported feeling “curious” (44.3%) significantly more often than Chinese participants (14.0%; χ^2^ = 11.843, *p* < 0.0015). In the medium condition, the negative emotion “uncomfortable” was reported by Koreans (41.0%) more frequently than by Chinese participants (4.0%; χ^2^ = 20.417, *p* < 0.0015). At the high spiciness level, significant differences emerged across multiple emotions. Koreans reported higher frequencies than their Chinese counterparts for positive emotions such as “curious” (χ^2^ = 11.336, *p* < 0.0015), “overjoyed” (χ^2^ = 12.953, *p* < 0.0015), and “surprised” (χ^2^ = 11.182, *p* < 0.0015). Similarly, for negative emotions, Koreans exhibited higher response frequencies for “fearful” (χ^2^ = 14.312, *p* < 0.0015), “nervous” (χ^2^ = 11.468, *p* < 0.0015), “pained” (χ^2^ = 12.308, *p* < 0.0015), and “uncomfortable” (χ^2^ = 16.077, *p* < 0.0015).

Figure 1 displays exploratory PCA biplots of the emotional responses of Korean (a) and Chinese (b) consumers to Xianglajiang, illustrating the relationships between spiciness levels and emotion descriptors.

For the Korean participants (Figure 1a), principal components 1 and 2 (PC1 and PC2) explained 69.6% and 27.2% of the total variance, respectively, accounting for a cumulative 96.8%. The three spiciness conditions formed a fan-shaped pattern, primarily along the negative side of PC1. The Mild condition was located in the lower-left quadrant (negative PC1 and PC2), the Medium condition was closer to the negative PC1 axis, and the High condition extended into the upper-left quadrant (negative PC1 and positive PC2). Emotional attributes 29 (Satisfied) and 13 (Expectant) were positioned near the Mild vector, while 7 (Curious) and 18 (Interested) appeared at the far lower-left edge of the plot. Attributes 32 (Uncomfortable), 1 (Agitated), and 8 (Disappointed) broadly aligned with the Medium vector. A compact cluster consisting of 12 (Excited), 22 (Overjoyed), 23 (Pained), and 30 (Surprised) was observed near the High vector. In contrast, the positive side of PC1 contained most of the remaining emotions, including 2 (Alert), 4 (Blissful), 5 (Calm), 6 (Comfortable), 9 (Disgusted), 10 (Eager), 11 (Energetic), 14 (Fearful), 15 (Friendly), 16 (Glad), 17 (Happy), 24 (Peaceful), 25 (Pleasant), 26 (Regret), 27 (Relaxed), 28 (Sad), 31 (Tender), and 33 (Worried).

For the Chinese data presented in Figure 1b, PC1 and PC2 accounted for 52.7% and 36.9% of the variance, respectively, leading to a cumulative total of 89.6%. The Mild and Medium vectors extended horizontally into the negative PC1 region, forming a narrow acute angle. In contrast, the High vector projected diagonally into the upper-right quadrant (positive PC1 and positive PC2), indicating a clear spatial separation from the other two conditions. Emotions 4 (Blissful), 5 (Calm), 6 (Comfortable), 17 (Happy), 24 (Peaceful), and 25 (Pleasant) were found within or near the space between the Mild and Medium vectors. Below the negative PC1 axis, a downward-trending cluster included emotions 16 (Glad), 20 (Joyful), 27 (Relaxed), 29 (Satisfied), and 31 (Tender). Along the High vector, emotions 1 (Agitated), 9 (Disgusted), 11 (Energetic), 21 (Nervous), and 26 (Regret) were arranged sequentially, while 19 (Irritated) was isolated at the upper end of the positive PC2 axis. Near the origin, a compact cluster comprised emotions 2 (Alert), 7 (Curious), 8 (Disappointed), 10 (Eager), 12 (Excited), 13 (Expectant), 15 (Friendly), 18 (Interested), 22 (Overjoyed), and 30 (Surprised), while emotions 28 (Sad) and 33 (Worried) were positioned on the lower-right periphery.

### 3.3. Group Differences in Emotional Responses to Xianglajiang by Spiciness Level

To comparatively explore the emotional responses of Korean and Chinese participants to different spiciness levels (mild, medium, and high) of Xianglajiang, a two-way mixed ANOVA was conducted for each variable. The results are summarized in Table 3, Table 4 and Table 5, with descriptive statistics provided in Table 3.

The two-way ANOVA indicated significant main effects for both nationality and spiciness intensity on emotional responses (*p* < 0.05). Notably, Korean participants reported higher baseline levels of negative emotions than Chinese participants across all spiciness levels (F_(1, 109)_ = 8.77, *p* < 0.01, ηp^2^ = 0.12). Additionally, a significant main effect of spiciness level on negative emotions was observed (F_(1.54, 167.78)_ = 29.38, *p* < 0.001, ηp^2^ = 0.21), with both groups exhibiting a consistent increase in negative affect as spiciness intensified from medium to high (Koreans: 1.21 ± 1.12; Chinese: 0.70 ± 0.80). Bonferroni-adjusted pairwise comparisons showed that positive emotion scores differed significantly only between the mild and high spiciness conditions (mean difference = 0.196, 95% CI [0.033, 0.359], *p* = 0.013), whereas no significant differences were observed between the mild and medium (*p* = 0.213) or medium and high (*p* = 0.480) conditions.

For positive emotions, a significant main effect of spiciness level was observed (F_(1.92, 209.63)_ = 4.95, *p* < 0.01, ηp^2^ = 0.04), as illustrated in Table 3. Positive emotions decreased with increasing spiciness, especially among Chinese participants (from 0.74 ± 0.92 at the mild level to 0.41 ± 0.67 at the high level). Bonferroni-adjusted pairwise comparisons indicated that negative emotion scores were significantly higher under the high-spiciness condition than under both the mild (mean difference = −0.553, 95% CI [−0.784, −0.323], *p* < 0.001) and medium conditions (mean difference = −0.465, 95% CI [−0.651, −0.280], *p* < 0.001). No significant difference was observed between the mild and medium conditions (*p* = 0.382). Nonetheless, no significant main effect of nationality was established for positive emotions (F_(1, 109)_ = 0.07, *p* > 0.05, ηp^2^ = 0.001).

Importantly, no significant interaction effects between spiciness level and nationality were detected for either positive (F_(1.92, 209.63)_ = 2.27, *p* > 0.05, ηp^2^ = 0.02) or negative (F_(1.54, 167.78)_ = 1.75, *p* > 0.05, ηp^2^ = 0.02) emotions (Table 4 and Table 5).

**Table 3:** Emotional Responses to Spiciness Levels.

		Korean (*n* = 61)	Chinese (*n* = 50)	*t*
Mild	Positive Emotion	0.67 ± 0.74 (0.40)	0.74 ± 0.92 (0.40)	−0.436
Medium		0.58 ± 0.72 (0.30)	0.59 ± 0.74 (0.38)	−0.120
High		0.60 ± 0.66 (0.45)	0.41 ± 0.67 (0.15)	1.457
Mild	Negative Emotion	0.52 ± 0.70 (0.33)	0.28 ± 0.59 (0.00)	1.941
Medium		0.63 ± 0.67 (0.42)	0.35 ± 0.55 (0.00)	2.413 *
High		1.21 ± 1.12 (0.83)	0.70 ± 0.80 (0.42)	2.708 **

M ± SD (Median), * *p* < 0.05, ** *p* < 0.01. Respondents rated intensity on a scale from 0 (not selected) to 5 (very strong): 1 = very weak, 3 = neutral, and 5 = very strong.

**Table 4:** Comparative Analysis of National Differences in Positive Emotions by Xianglajiang Spiciness Level.

	SS	*df*	MS	*F*	ηp^2^
Within					
Spiciness level	2.13	1.92	1.11	4.95 **	0.04
Spiciness × Nationality	0.98	1.92	0.51	2.27	0.02
Error (Spiciness)	46.90	209.63	0.22		
Between					
Nationality	0.09	1	0.09	0.07	0.001
Error	135.36	109	1.24		

** *p* < 0.01. SS = Sum of Squares; *df* = degrees of freedom; MS = Mean Square; *F* = *F*-statistic.

**Table 5:** Comparative Analysis of National Differences in Negative Emotions by Xianglajiang Spiciness Level.

	SS	*df*	MS	*F*	ηp^2^
Within					
Spiciness level	19.42	1.54	12.63	29.38 ***	0.21
Spiciness × Nationality	1.16	1.54	0.75	1.75	0.02
Error (Spiciness)	72.13	167.78	0.43		
Between					
Nationality	9.84	1	9.84	8.77 **	0.12
Error	122.39	109	1.12		

** *p* < 0.01, *** *p* < 0.001. SS = Sum of Squares; *df* = degrees of freedom; MS = Mean Square; *F* = *F*-statistic.

### 3.4. Emotional Responses by Individual Characteristics

Analysis of sensation-seeking tendencies by nationality revealed that Chinese participants generally scored higher than their Korean counterparts. Significant differences emerged in several categories, including TAS (TAS1: *t* = −4.899, *p* < 0.001; TAS3: *t* = −2.381, *p* < 0.05), ES (ES3: *t* = −2.071, *p* < 0.05), DIS (DIS1: *t* = −2.236, *p* < 0.05), and BS (BS2: *t* = −2.790, *p* < 0.01), with Chinese participants reporting significantly higher scores in these areas (Table 6).

### 3.5. Correlation Analysis of Preference, Attitude, and Purchase Intention by Spiciness Level for Xianglajiang

Table 7, Table 8 and Table 9 summarize the correlation analysis of sensory attributes by spiciness level for Xianglajiang. In the mild condition, positive emotions correlated significantly with preference (r = 0.280, *p* < 0.01), attitude (r = 0.308, *p* < 0.01), and purchase intention (r = 0.236, *p* < 0.05). Conversely, negative emotions exhibited significant negative correlations with preference (r = −0.270, *p* < 0.01), attitude (r = −0.245, *p* < 0.01), and purchase intention (r = −0.343, *p* < 0.01). In the medium condition, positive emotions displayed stronger positive correlations with preference (r = 0.416, *p* < 0.01), attitude (r = 0.464, *p* < 0.01), and purchase intention (r = 0.332, *p* < 0.01). In contrast, negative emotions again negatively correlated with preference (r = −0.313, *p* < 0.01), attitude (r = −0.281, *p* < 0.01), and purchase intention (r = −0.387, *p* < 0.01). For the high condition, positive emotions positively correlated with preference (r = 0.351, *p* < 0.01), attitude (r = 0.355, *p* < 0.01), and purchase intention (r = 0.308, *p* < 0.01), though these correlations were slightly weaker than in the medium condition. Negative emotions demonstrated significant negative correlations with preference (r = −0.367, *p* < 0.01), attitude (r = −0.359, *p* < 0.01), and purchase intention (r = −0.400, *p* < 0.01).

## 4. Discussion

This study explored emotional responses to varying spiciness levels in Xianglajiang, a traditional Chinese spicy sauce, and assessed the impact of national background and sensation-seeking tendencies among Korean and Chinese consumers. The findings indicated that negative emotions increased significantly with higher spiciness, while positive emotions remained relatively stable or slightly decreased. Korean consumers generally reported greater levels of negative emotions than their Chinese counterparts across all spiciness levels. Both groups experienced a significant rise in negative emotions as spiciness intensified from mild to high.

Analysis of individual emotion descriptors indicated that Korean consumers reported higher frequencies of both positive (e.g., curious, surprised, and overjoyed) and negative (e.g., fearful, nervous, pained, and uncomfortable) high-spiciness conditions. PCA further revealed distinct emotional response patterns based on nationality and spiciness intensity. Chinese consumers displayed more familiar and neutral emotional responses under mild and medium spiciness, while stronger negative emotions emerged in high-spiciness conditions. Additionally, Chinese participants exhibited higher scores in sensation-seeking tendencies than their Korean counterparts, suggesting that individual differences in sensation-seeking may contribute to variations in the acceptance of intense sensory experiences. Across all spiciness levels, positive emotions positively correlated with preference, attitude, and purchase intention, whereas negative emotions exhibited inverse relationships with these consumer acceptance measures.

Across all spiciness levels, positive emotions positively correlated with preference, attitude, and purchase intention, whereas negative emotions exhibited inverse relationships with these consumer acceptance measures. The positive association between positive and negative emotion scores across all spiciness levels suggests that consumers may simultaneously experience both positive and negative emotions when consuming spicy foods. This emotional ambivalence indicates that positive and negative emotions are not necessarily mutually exclusive in response to spicy foods. In addition, the strong positive correlations among preference, attitude, and purchase intention indicate that these consumer acceptance measures are closely related.

Overall, emotional responses to Xianglajiang were shaped by spiciness intensity, cultural familiarity, and individual sensation-seeking tendencies. These findings enhance our understanding of cross-national consumer reactions to traditional Chinese spicy sauces and provide practical implications for developing and localizing spicy food products in global markets.

### 4.1. Cultural Familiarity and Emotional Response Mechanism

In this study, Chinese consumers exhibited more positive and stable emotional responses to Xianglajiang than Korean consumers, particularly under mild and medium spiciness conditions. Conversely, Korean consumers reported higher levels of negative emotions toward the sauce. These results suggest that cultural familiarity considerably influences the direction and intensity of emotional responses to spicy foods.

Research indicates that food familiarity extends beyond simple repeated exposure and is closely associated with expectation formation, sensory prediction, and overall hedonic evaluations [27]. Familiar foods allow consumers to interpret sensory information efficiently based on prior experiences and cultural knowledge, mitigating discrepancies between expected and actual sensory experiences and fostering stable, positive emotional responses [28]. Furthermore, emotional responses to foods are not merely direct reactions to sensory stimuli but are shaped through interpretation and meaning-making processes, with familiar foods more likely to elicit automatic, positively valenced emotional associations [29].

Conversely, unfamiliar foods may not align with established memory-based expectations, increasing cognitive processing demands and uncertainty during sensory evaluation. Consequently, such foods may be perceived as less predictable or potentially risky [30], leading to stronger negative emotional responses. This phenomenon is closely related to food neophobia and uncertainty-driven affective reactions.

From this perspective, Xianglajiang—a traditional spicy sauce prevalent in Chinese food culture—might have functioned as a culturally familiar stimulus for Chinese consumers, aligning closely with their sensory expectations. Consequently, they reported more positive and neutral emotional responses under mild and medium spiciness conditions. In contrast, Korean consumers, less familiar with Xianglajiang’s unique spice profile and sensory attributes, likely experienced greater cognitive effort during sensory evaluation, contributing to the more pronounced negative emotional responses observed among them.

### 4.2. Flavor Characteristics of Xianglajiang and Emotional Responses

Xianglajiang, a traditional spicy sauce widely used in Sichuan cuisine, features a complex sensory profile that extends beyond capsaicin-induced pungency. In particular, the numbing effect of huajiao (Sichuan pepper), combined with the aromatic richness of various spices, creates a distinctive sensory experience [31,32]. Therefore, Xianglajiang offers a multilayered flavor profile that encompasses heat, aroma, and trigeminal stimulation.

Food-evoked emotional responses arise not from a single sensory attribute but from the interplay of multiple flavor components, including aroma, taste, texture, and other sensory characteristics [33]. These flavor combinations strongly influence consumers’ expectations and sensory interpretations, ultimately shaping their emotional responses and preferences. Consequently, emotional reactions are more closely linked to the alignment between sensory experiences and prior cultural exposure than to the physical intensity of the stimulus itself.

From this perspective, the notable cross-national differences observed in emotional responses to Xianglajiang can be attributed not only to variations in familiarity but also to the interaction between the cultural specificity of each sauce’s flavor profile and the level of congruence between sensory expectations and experiences. This suggests that emotional reactions to food are more influenced by overall flavor architecture and culturally learned sensory experiences than by spiciness alone.

Ultimately, these insights emphasize that consumers’ emotional experiences are shaped by the integrated sensory characteristics of a product, rather than solely by pungency intensity. This underscores the importance of considering the overall flavor architecture in the development of spicy food products.

### 4.3. Consumers’ Ambivalent Emotional Responses to Spicy Stimuli

In this study, Korean consumers displayed ambivalent emotional responses to high-spiciness Xianglajiang, experiencing both positive and negative emotions simultaneously. Participants reported higher frequencies of positive emotions such as ‘curious’, ‘surprised’, and ‘overjoyed’ and negative emotions such as ‘fearful’, ‘nervous’, ‘pained’, and ‘uncomfortable’. These findings indicate that intense spiciness is not purely negative; rather, it can represent a complex emotional experience where pleasure and discomfort coexist [33].

This pattern can be explained from the perspective of hedonic response. While highly spicy foods may cause physical discomfort, they can also evoke excitement, interest, and enjoyment from intense sensory stimulation. Therefore, high spiciness may serve as a powerful trigger for both negative emotions and positive arousal. This phenomenon can also be interpreted through the concept of benign masochism, where intense sensory experiences, such as consuming spicy food, initially cause discomfort or pain but are subsequently reappraised as safe and enjoyable [34]. Consequently, consumers are less likely to view the stimulus as threatening and instead perceive it as a challenging yet rewarding sensory experience.

These findings align with previous research suggesting that consuming spicy foods often elicits both pleasure and discomfort. While earlier studies focused on theoretical or psychological viewpoints, this study offers empirical evidence that positive and negative emotions can coexist when consuming a culturally unfamiliar spicy sauce. This indicates that consumers’ emotional responses to spicy foods should be viewed as multidimensional experiences, shaped by both sensory stimulation and cultural context.

This complexity is further reflected in the methodological divergence observed in our results. Although the aggregate emotional scores from the two-way ANOVA yielded no significant interaction between nationality and spiciness intensity—indicating a common trend in emotional shifts—the individual emotion frequencies (RATA) revealed distinct qualitative profiles between the two cultural groups. Notably, under high spiciness conditions, Korean consumers reported significantly higher frequencies of specific ambivalent emotions, such as curiosity and fear. This suggests that while the overall intensity of negative emotions increases uniformly with sensory heat across nationalities, the qualitative nuance and discrete emotional responses triggered by culturally unfamiliar spicy stimuli are heavily influenced by cultural background. Therefore, evaluating both aggregate emotional dimensions and discrete emotional terms offers a more comprehensive and holistic understanding of cross-cultural consumer experiences.

### 4.4. Sensation-Seeking Tendencies and Cross-National Differences

Chinese consumers exhibited a higher sensation-seeking tendency than their Korean counterparts. This difference potentially emanates from cross-cultural variations in dietary environments and sensory exposure in food contexts. In particular, Chinese dietary culture features a wide range of regional cuisines and frequent encounters with complex and intense sensory profiles [35]. Such exposure likely fosters greater familiarity with strong and varied sensory stimulation in food-related contexts.

Accordingly, a higher sensation-seeking tendency may reflect accumulated exposure to diverse sensory food experiences rather than inherent cultural or dispositional differences. However, this interpretation should be viewed as associative since sensation-seeking is influenced by various psychological and environmental factors.

Conversely, the lower sensation-seeking tendency observed among Korean consumers may be linked to less frequent exposure to strongly spiced flavors, such as Xianglajiang, in their daily diets. These differences in sensory exposure history may affect responsiveness to novel or intense stimuli, potentially leading to cross-national variations in emotional responses and acceptance of spicy foods.

### 4.5. Limitations

Despite its contributions, this study has certain limitations. First, the sample size was relatively limited and exclusively comprised Korean and Chinese participants, which may have reduced the statistical power of the analyses and restricted the generalizability of the findings to other cultural groups. Nevertheless, the present findings provide valuable preliminary evidence that can inform future studies with larger and more diverse samples. Future research should include a broader range of nationalities to provide deeper insights into cross-cultural emotional responses to spicy foods.

Second, emotional responses were assessed through self-reported questionnaires, which can be subject to personal interpretation and response bias. Future research could enhance these findings by incorporating physiological or biometric measures, such as facial expression analysis, heart rate variability, or skin conductance responses, to provide a more comprehensive assessment of consumer emotions.

Third, this study concentrated on emotional responses to a specific traditional Chinese spicy sauce, Xianglajiang. While Xianglajiang is a culturally unique condiment, the results may not apply to other spicy food products with different flavor profiles or sensory characteristics. In addition, Xianglajiang includes various sensory attributes beyond spiciness, such as aromatic spices and the numbing sensation from huajiao (Sichuan pepper). In addition, quantitative information on the proportions of individual ingredients in commercial Xianglajiang products is generally unavailable. Therefore, only the typical ingredients and production characteristics could be described, which may limit a more detailed interpretation of the relationship between ingredient composition and the observed emotional responses. Therefore, the emotional responses identified in this study may reflect a combination of multiple sensory characteristics rather than spiciness intensity alone. Future research should explore a broader range of spicy foods and condiments to better understand how spiciness, aroma, and other flavor attributes contribute to emotional responses and consumer acceptance.

Fourth, this study focused on immediate emotional responses during a single tasting session. Longitudinal studies examining repeated exposure, familiarity development, and actual consumption behavior would provide a more comprehensive understanding of the factors influencing consumer acceptance of spicy foods.

Finally, emotional responses were aggregated into positive and negative dimensions for statistical analysis. While this simplification aided interpretation, it might have obscured the complex relationships among individual emotions. Future studies could employ advanced analytical techniques, such as emotion network analysis, cluster analysis, or latent profile analysis, to better understand the multidimensional nature of emotional experiences associated with spicy food consumption. In addition, Face tracking during evaluation could provide complementary real-time, objective measures of emotional responses, thereby enriching the interpretation of self-reported emotional data.

### 4.6. Practical Implications

The present findings have significant implications for the product development, localization, and commercialization of spicy foods in international markets. Our results indicate that consumers’ emotional responses to spicy products are influenced not only by sensory intensity but also by cultural familiarity and individual sensation-seeking tendencies. Therefore, product development strategies should go beyond merely adjusting pungency and incorporate consumers’ emotional experiences as a core component of product optimization. Emotional profiling during new product development can help manufacturers identify flavor formulations that satisfy sensory expectations while fostering positive emotional connections with target consumers.

Furthermore, our data suggest that localization strategies must be customized to the unique characteristics of individual markets. For instance, while Korean consumers exhibited stronger negative emotional responses under high-spiciness conditions, they also reported positive feelings such as curiosity and surprise. This emotional profile suggests that Korean consumers do not merely find highly spicy products unpleasant; they also view them as both appealing and challenging. Consequently, instead of drastically reducing the overall sensory impact of Xianglajiang, manufacturers could benefit from creating a product portfolio with varying spiciness levels. This approach would allow consumers to acclimate gradually while maintaining the product’s distinctive sensory identity, thereby reducing psychological barriers to unfamiliar foods without compromising authenticity.

Xianglajiang presents a unique sensory profile that includes capsaicin-induced heat, aromatic complexity, and the numbing sensation characteristic of huajiao (Sichuan pepper). Thus, effective localization requires a careful balance of these attributes based on market-specific preferences. Rather than modifying a single sensory parameter, developers should harmonize the overall sensory experience while preserving the core flavor characteristics that define the product. This highlights the importance of considering a food’s integrated flavor architecture during reformulation for global markets.

In contrast, Chinese consumers demonstrated consistent emotional responses across all spiciness levels and exhibited a greater tendency for sensation-seeking than Korean consumers. These findings indicate that preserving the authentic sensory characteristics of Xianglajiang is a more effective strategy for the domestic Chinese market than extensive modifications. Instead of diluting sensory intensity, product developers should emphasize product authenticity through traditional recipes, regional identity, and premium quality attributes, thereby reinforcing consumers’ emotional attachment to culturally familiar products.

These insights also inform strategic marketing communications. The emotional profiles derived from this study provide a robust framework for designing market-specific promotional campaigns. For instance, marketing efforts aimed at Korean consumers should emphasize curiosity, sensory exploration, and the unique opportunity to experience authentic Chinese flavors while addressing perceived uncertainty through adjustable spiciness options or recommended serving pairings. Conversely, promotional strategies for the Chinese market should focus on authenticity, culinary heritage, and the traditional cultural values inherent in Xianglajiang. Such tailored communication can enhance pre-consumption emotional expectations and improve product acceptance.

Generally, this study illustrates that emotional profiling can serve as a strategic decision-making tool throughout the product development lifecycle. While traditional sensory evaluations often rely on hedonic measures such as liking, preference, and purchase intent, our research demonstrates that discrete emotional responses provide deeper psychological insights that hedonic scales alone cannot capture. Incorporating emotional assessments into standard sensory testing allows manufacturers to identify drivers of consumer rejection, optimize flavor formulations, and refine product positioning before commercialization. Ultimately, this emotion-based framework establishes a systematic approach for the successful localization and global expansion of culturally distinct spicy food products such as Xianglajiang.

## 5. Conclusions

This study investigated emotional responses to varying levels of spiciness in Xianglajiang among Korean and Chinese consumers, considering the impact of national background and sensation-seeking tendencies. The results indicated that negative emotions increased with spiciness intensity, while positive emotions remained relatively stable or declined. Korean consumers generally reported higher levels of negative emotions than their Chinese counterparts across all spiciness levels, with both groups experiencing a marked increase in negative affect as spiciness intensified. In addition, significant cross-national differences in specific emotional responses may reflect differences in cultural familiarity related to spicy foods.

Furthermore, the findings reveal a significant link between emotional responses and consumer acceptance measures, including preference, attitude, and purchase intention. Positive emotions generally correlated with favorable consumer evaluations, while negative emotions were associated with lower acceptance levels. Additionally, a tendency for sensation-seeking emerged as a key individual characteristic that may contribute to variability in reactions to intense spiciness.

Overall, these results indicate that consumer acceptance of Xianglajiang is associated with spiciness intensity, and the observed cross-national differences may reflect differences in cultural familiarity, together with individual sensation-seeking tendencies. This research enhances our understanding of cross-national emotional responses to traditional Chinese spicy sauces and offers practical insights for developing and localizing spicy food products in global markets.

From an industrial standpoint, these findings underline that successful localization of spicy food products requires more than merely adjusting spiciness levels. Product development strategies should also consider consumers’ emotional responses, cultural familiarity, and individual differences in sensation-seeking tendencies to optimize product acceptance across various markets. Such emotion-based localization strategies may facilitate the successful global expansion of traditional spicy condiments such as Xianglajiang.

Future research should extend this framework by including consumers from diverse cultural backgrounds and exploring a wider array of spicy foods and condiments. Additionally, studies that incorporate different food carriers and repeated consumption contexts could yield a more comprehensive understanding of consumer acceptance and emotional responses to spicy foods.

## Figures and Tables

**Figure 1:**
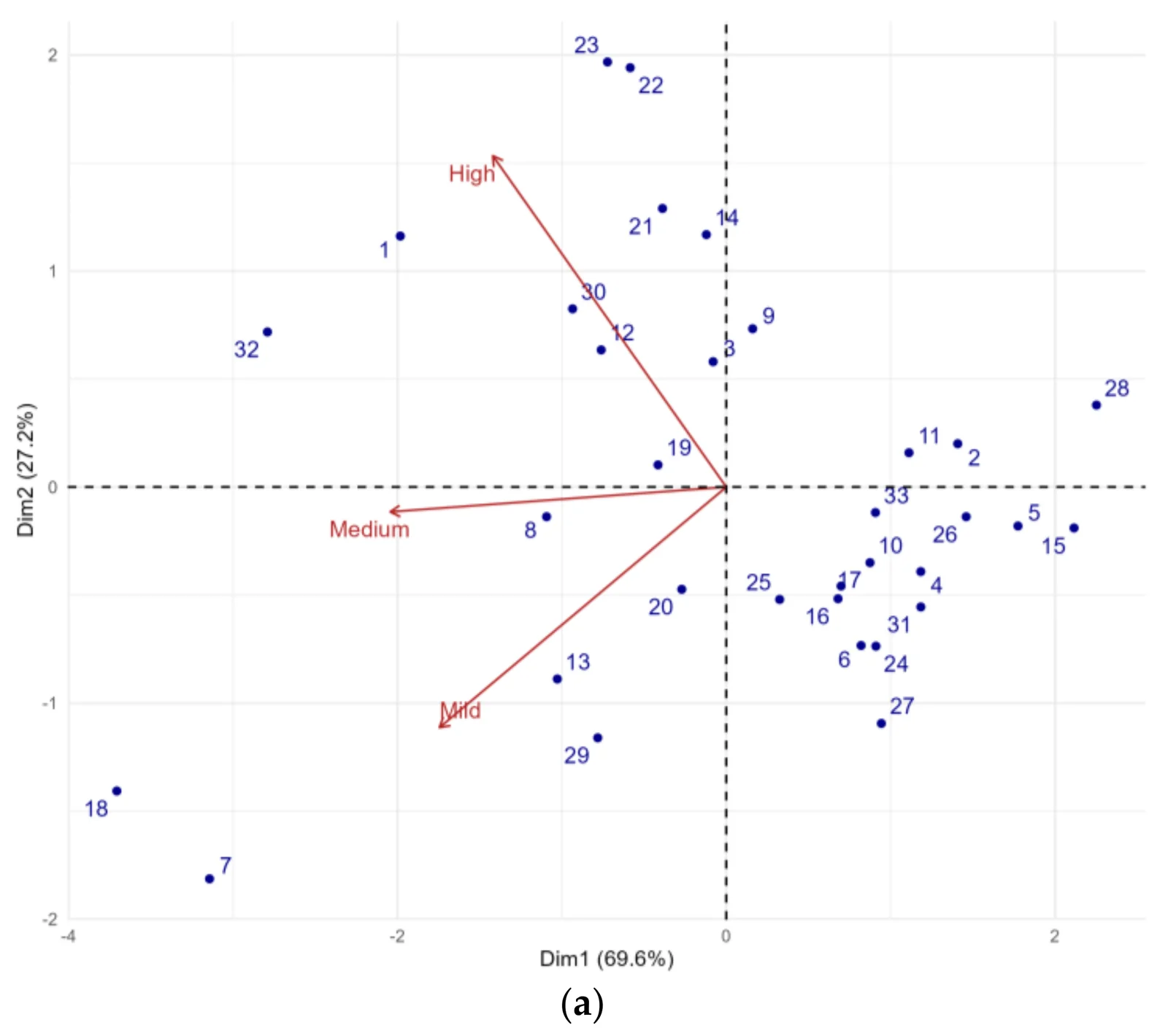

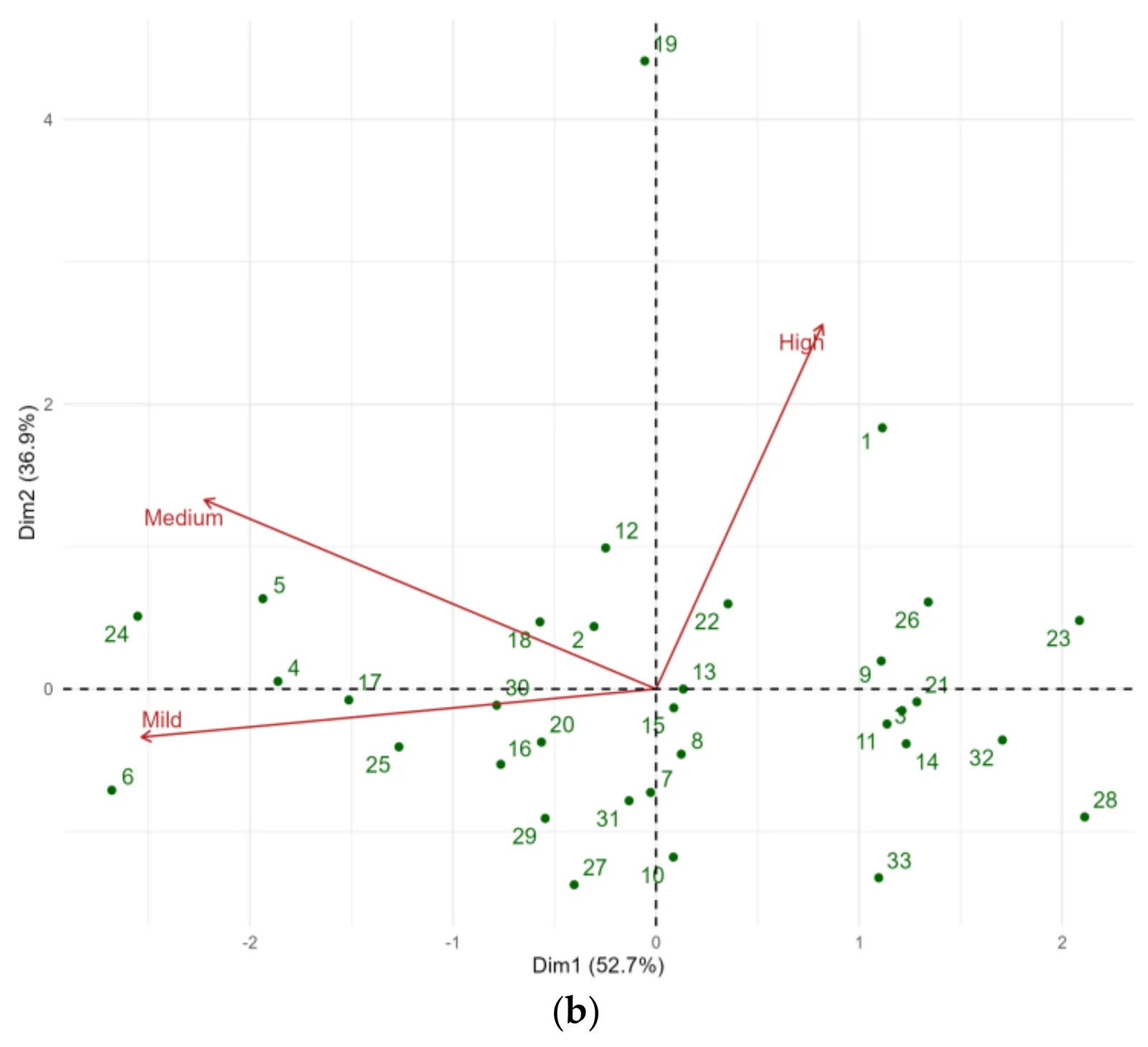
PCA Biplots of Emotional Responses to Xianglajiang by Spiciness Level. (**a**): Korean, (**b**): Chinese. Emotional descriptors: 1 = Alert, 2 = Blissful, 3 = Calm, 4 = Comfortable, 5 = Curious, 6 = Eager, 7 = Energetic, 8 = Excited, 9 = Expectant, 10 = Friendly, 11 = Glad, 12 = Happy, 13 = Interested, 14 = Joyful, 15 = Overjoyed, 16 = Peaceful, 17 = Pleasant, 18 = Relaxed, 19 = Satisfied, 20 = Surprised, 21 = Tender, 22 = Agitated, 23 = Anxious, 24 = Disappointed, 25 = Disgusted, 26 = Fearful, 27 = Irritated, 28 = Nervous, 29 = Pained, 30 = Regret, 31 = Sad, 32 = Uncomfortable, 33 = Worried.

**Table 1:** Demographics of Study Participants.

		Total (*N* = 111)	Korean (*n* = 61)	Chinese (*n* = 50)	Test Statistic
Sex	Male	55 (49.5)	30 (49.2)	25 (50.0)	0.007
	Female	56 (50.5)	31 (50.8)	25 (50.0)	
Education	High school graduate	17 (15.3)	8 (13.1)	9 (18.0)	10.747 *
	Associate’s degree	23 (20.7)	7 (11.5)	16 (32.0)	
	Bachelor’s degree	46 (41.1)	27 (44.3)	19 (38.0)	
	Graduate degree	25 (22.5)	19 (31.1)	6 (12.0)	
Occupation	Student	24 (21.6)	8 (13.1)	16 (32.0)	14.979
	Homemaker	9 (8.1)	6 (9.8)	3 (6.0)	
	Office worker	22 (19.8)	14 (23.0)	8 (16.0)	
	Public official	4 (3.6)	1 (1.6)	3 (6.0)	
	Self-employed	8 (7.2)	4 (6.6)	4 (8.0)	
	Professional	9 (8.1)	7 (11.5)	2 (4.0)	
	Service worker	18 (16.2)	13 (21.3)	5 (10.0)	
	Production worker	3 (2.7)	0 (0.0)	3 (6.0)	
	Other	14 (12.6)	8 (13.1)	6 (12.0)	
Spicy sauce consumption frequency	Once every 2–3 months	12 (10.8)	9 (14.8)	3 (6.0)	9.045
	Once a month	17 (15.3)	12 (19.7)	5 (10.0)	
	2–3 times a month	19 (17.1)	10 (16.4)	9 (18.0)	
	1–2 times a week	26 (23.4)	13 (21.3)	13 (26.0)	
	3–4 times a week	25 (22.5)	10 (16.4)	15 (30.0)	
	Once a day	9 (8.1)	4 (6.6)	5 (10.0)	
	2–3 times a day	3 (2.7)	3 (4.9)	0 (0.0)	
Preference for spicy sauces	Strongly dislike	5 (4.5)	5 (8.2)	0 (0.0)	20.582 ***
	Dislike	16 (14.4)	15 (24.6)	1 (2.0)	
	Neutral	32 (28.8)	11 (18.0)	21 (42.0)	
	Like	42 (37.8)	20 (32.8)	22 (44.0)	
	Strongly like	16 (14.4)	10 (16.4)	6 (12.0)	
Age		38.18 ± 12.49	39.74 ± 11.61	36.28 ± 13.36	0.833
Preference for spicy sauces		3.43 ± 1.05	3.25 ± 1.12	3.66 ± 0.72	−2.099 *
Sensation-seeking		3.22 ± 0.72	3.09 ± 0.85	3.37 ± 0.47	−2.122 *

Values are presented as N (%) or the mean (M) ± standard deviation (SD). Categorical and continuous variables were compared using χ^2^ and independent *t*-tests, respectively. * *p* < 0.05, *** *p* < 0.001.

**Table 2:** Emotional Response Frequency.

Emotion		Mild			Medium			High		
		Korean (*n* = 61)	Chinese (*n* = 50)	χ^2^	Korean (*n* = 61)	Chinese (*n* = 50)	χ^2^	Korean (*n* = 61)	Chinese (*n* = 50)	χ^2^
Positive	Alert	6 (9.8)	12 (24.0)	4.057	8 (13.1)	7 (14.0)	0.018	8 (13.1)	9 (18.0)	0.506
	Blissful	11 (18.0)	15 (30.0)	2.194	9 (14.8)	10 (20.0)	0.533	7 (11.5)	5 (8.2)	0.062
	Calm	8 (13.1)	14 (28.0)	3.831	8 (13.1)	13 (26.0)	2.974	6 (9.8)	7 (14.0)	0.461
	Comfortable	13 (21.3)	16 (32.0)	1.626	11 (18.0)	12 (24.0)	0.596	6 (9.8)	1 (2.0)	2.856
	Curious	27 (44.3)	7 (14.0)	11.843 *	20 (32.8)	8 (16.0)	4.105	15 (24.6)	1 (2.0)	11.336 *
	Eager	9 (14.8)	9 (18.0)	0.213	10 (16.4)	3 (6.0)	2.871	7 (11.5)	3 (6.0)	1.005
	Energetic	7 (11.5)	9 (18.0)	0.948	9 (14.8)	1 (2.0)	5.453	10 (16.4)	8 (16.0)	0.003
	Excited	12 (19.7)	8 (16.0)	0.251	15 (24.6)	9 (18.0)	0.704	19 (31.1)	8 (16.0)	3.425
	Expectant	19 (31.1)	11 (22.0)	1.166	17 (27.9)	4 (8.0)	7.071	13 (21.3)	8 (16.0)	0.505
	Friendly	8 (13.1)	7 (14.0)	0.018	5 (8.2)	6 (12.0)	0.445	4 (6.6)	6 (12.0)	0.993
	Glad	11 (18.0)	9 (18.0)	0.000	10 (16.4)	7 (14.0)	0.121	7 (11.5)	3 (6.0)	1.005
	Happy	13 (21.3)	12 (24.0)	0.114	8 (13.1)	8 (16.0)	0.185	11 (18.0)	5 (10.0)	1.437
	Interested	29 (47.5)	10 (20.0)	9.145	23 (37.7)	8 (16.0)	6.431	19 (31.1)	8 (16.0)	3.425
	Joyful	14 (23.0)	11 (22.0)	0.014	15 (24.6)	6 (12.0)	2.839	11 (18.0)	5 (10.0)	1.437
	Overjoyed	9 (14.8)	5 (10.0)	0.563	13 (21.3)	8 (16.0)	0.505	28 (45.9)	7 (14.0)	12.953 *
	Peaceful	15 (24.6)	15 (30.0)	0.408	10 (16.4)	14 (28.0)	2.184	7 (11.5)	5 (10.0)	0.062
	Pleasant	12 (19.7)	11 (22.0)	0.091	13 (21.3)	9 (18.0)	0.190	9 (14.8)	3 (6.0)	2.184
	Relaxed	15 (24.6)	10 (20.0)	0.332	7 (11.5)	5 (10.0)	0.062	5 (8.2)	1 (2.0)	2.064
	Satisfied	21 (34.4)	11 (22.0)	2.068	18 (29.5)	5 (10.0)	6.366	12 (19.7)	3 (6.0)	4.395
	Surprised	13 (21.3)	10 (20.0)	0.029	14 (23.0)	7 (14.0)	1.435	23 (37.7)	5 (10.0)	11.182 *
	Tender	12 (19.7)	6 (12.0)	1.190	9 (14.8)	7 (14.0)	0.013	6 (9.8)	2 (4.0)	1.399
Negative	Agitated	15 (24.6)	5 (10.0)	3.960	19 (31.1)	8 (16.0)	3.425	32 (52.5)	14 (28.0)	6.774
	Anxious	10 (16.4)	5 (10.0)	0.961	12 (19.7)	5 (10.0)	1.982	17 (27.9)	6 (12.0)	4.212
	Disappointed	13 (21.3)	7 (14.0)	0.994	16 (26.2)	8 (16.0)	1.697	15 (24.6)	3 (6.0)	6.989
	Disgusted	10 (16.4)	6 (12.0)	0.430	11 (18.0)	5 (10.0)	1.437	20 (32.8)	9 (18.0)	3.113
	Fearful	12 (19.7)	3 (6.0)	4.395	13 (21.3)	4 (8.0)	3.754	24 (39.3)	4 (8.0)	14.312 *
	Irritated	13 (21.3)	7 (14.0)	0.994	14 (23.0)	13 (26.0)	0.139	15 (24.6)	22 (44.0)	4.658
	Nervous	12 (19.7)	4 (8.0)	3.034	13 (21.3)	4 (8.0)	3.754	25 (41.0)	6 (12.0)	11.468 *
	Pained	11 (18.0)	3 (6.0)	3.610	14 (23.0)	3 (6.0)	6.087	32 (52.5)	10 (20.0)	12.308 *
	Regret	9 (14.8)	7 (14.0)	0.013	5 (8.2)	3 (6.0)	0.198	9 (14.8)	9 (18.0)	0.213
	Sad	5 (8.2)	4 (8.0)	0.001	5 (8.2)	0 (0.0)	4.292	6 (9.8)	5 (10.0)	0.001
	Uncomfortable	19 (31.1)	4 (8.0)	8.962	25 (41.0)	2 (4.0)	20.417 *	29 (47.5)	6 (12.0)	16.077 *
	Worried	11 (18.0)	7 (14.0)	0.329	11 (18.0)	2 (4.0)	5.233	9 (14.8)	2 (4.0)	3.559

*n* (%), * *p* < 0.0015 (Bonferroni correction).

**Table 6:** Comparison of Sensation-seeking Tendency by Nationality.

		Total (*N* = 111)	Korean (*n* = 61)	Chinese (*n* = 50)	*t*
TAS	TAS1	2.86 ± 1.20	2.41 ± 1.24	3.40 ± 0.88	−4.899 ***
	TAS2	3.22 ± 1.10	3.18 ± 1.26	3.26 ± 0.88	−0.392
	TAS3	3.16 ± 1.18	2.93 ± 1.34	3.44 ± 0.88	−2.381 *
ES	ES1	3.68 ± 1.03	3.61 ± 1.14	3.76 ± 0.87	0.802
	ES2	3.05 ± 1.12	2.95 ± 1.31	3.18 ± 0.82	−1.122
	ES3	3.20 ± 1.17	3.00 ± 1.34	3.44 ± 0.88	−2.071 *
DIS	DIS1	2.71 ± 1.04	2.52 ± 1.22	2.94 ± 0.71	−2.236 *
	DIS2	3.32 ± 1.04	3.36 ± 1.14	3.28 ± 0.90	0.415
	DIS3	3.19 ± 1.17	3.20 ± 1.31	3.18 ± 0.98	0.077
BS	BS1	3.42 ± 0.96	3.36 ± 1.02	3.50 ± 0.89	−0.771
	BS2	3.47 ± 1.07	3.23 ± 1.22	3.76 ± 0.77	−2.790 **
	BS3	3.30 ± 1.10	3.28 ± 1.25	3.32 ± 0.89	−0.202

M ± SD, * *p* < 0.05, ** *p* < 0.01, *** *p* < 0.001.

**Table 7:** Correlation Analysis of Sensory Attributes of Mild Xianglajiang.

	Positive Emotion	Negative Emotion	Preference	Attitude	Purchase Intention
1					
2	0.438 **				
3	0.280 **	−0.270 **			
4	0.308 **	−0.245 **	0.873 **		
5	0.236 *	−0.343 **	0.832 **	0.835 **	

* *p* < 0.05, ** *p* < 0.01. 1 = Positive Emotion; 2 = Negative Emotion; 3 = Preference; 4 = Attitude; 5 = Purchase Intention.

**Table 8:** Correlation Analysis of Sensory Attributes of Medium Xianglajiang.

	Positive Emotion	Negative Emotion	Preference	Attitude	Purchase Intention
1					
2	0.408 **				
3	0.416 **	−0.313 **			
4	0.464 **	−0.281 **	0.879 **		
5	0.332 **	−0.387 **	0.859 **	0.835 **	

** *p* < 0.01. 1 = Positive Emotion; 2 = Negative Emotion; 3 = Preference; 4 = Attitude; 5 = Purchase Intention.

**Table 9:** Correlation Analysis of Sensory Attributes of High Xianglajiang.

	Positive Emotion	Negative Emotion	Preference	Attitude	Purchase Intention
1					
2	0.315 **				
3	0.351 **	−0.367 **			
4	0.355 **	−0.359 **	0.868 **		
5	0.308 **	−0.400 **	0.831 **	0.889 **	

** *p* < 0.01. 1 = Positive Emotion; 2 = Negative Emotion; 3 = Preference; 4 = Attitude; 5 = Purchase Intention.

## Data Availability

The data presented in this study are available on request from the corresponding authors. The data are not publicly available due to privacy restrictions.

## Abbreviations

The following abbreviations are used in this manuscript:

TAS	Thrill and Adventure Seeking
ES	Experience Seeking
Dis	Disinhibition
BS	Boredom Susceptibility
